# Genome-wide identification of associations between enhancer and alternative splicing in human and mouse

**DOI:** 10.1186/s12864-022-08537-1

**Published:** 2022-05-09

**Authors:** Cheng-Kai Shiau, Jia-Hsin Huang, Yu-Ting Liu, Huai-Kuang Tsai

**Affiliations:** 1 Institute of Information Science, Academia Sinica, Taipei, 115 Taiwan; 2 Bioinformatics Program, International Graduate Program, Academia Sinica, Taipei, 115 Taiwan; 3 Institute of Biomedical Informatics, National Yang Ming Chiao Tung University, Taipei, 115 Taiwan

**Keywords:** Enhancer, Alternative splicing, Association analysis

## Abstract

**Background:**

Alternative splicing (AS) increases the diversity of transcriptome and could fine-tune the function of genes, so that understanding the regulation of AS is vital. AS could be regulated by many different *cis*-regulatory elements, such as enhancer. Enhancer has been experimentally proved to regulate AS in some genes. However, there is a lack of genome-wide studies on the association between enhancer and AS (enhancer-AS association). To bridge the gap, here we developed an integrative analysis on a genome-wide scale to identify enhancer-AS associations in human and mouse.

**Result:**

We collected enhancer datasets which include 28 human and 24 mouse tissues and cell lines, and RNA-seq datasets which are paired with the selected tissues. Combining with data integration and statistical analysis, we identified 3,242 human and 7,716 mouse genes which have significant enhancer-AS associations in at least one tissue. On average, for each gene, about 6% of enhancers in human (5% in mouse) are associated to AS change and for each enhancer, approximately one gene is identified to have enhancer-AS association in both human and mouse. We found that 52% of the human significant (34% in mouse) enhancer-AS associations are the co-existence of homologous genes and homologous enhancers. We further constructed a user-friendly platform, named Visualization of Enhancer-associated Alternative Splicing (VEnAS, http://venas.iis.sinica.edu.tw/), to provide genomic architecture, intuitive association plot, and contingency table of the significant enhancer-AS associations.

**Conclusion:**

This study provides the first genome-wide identification of enhancer-AS associations in human and mouse. The results suggest that a notable portion of enhancers are playing roles in AS regulations. The analyzed results and the proposed platform VEnAS would provide a further understanding of enhancers on regulating alternative splicing.

**Supplementary Information:**

The online version contains supplementary material available at 10.1186/s12864-022-08537-1.

## Background

Alternative splicing (AS) is one of the important processes during RNA maturation in higher eukaryotes. By including or excluding alternative exons, AS increases the diversity of downstream RNA products. More than 90% of genes with multiple exons undergo AS [1]. The inclusion and exclusion of exons by AS shape the downstream protein diversity [2]. Furthermore, AS participates in many key biological processes, such as developmental stages [3], tissue types [4, 5], genders [6, 7], insect caste determination [8], and so on. Thus, understanding the regulation of AS is vital.

The regulation of AS relies on numerous *cis*-regulatory elements, including *cis*-acting splicing regulatory elements (SREs), splicing motifs, and enhancers. SREs include exonic/intronic splicing enhancers or silencers. Wang et al. had conducted a systematical method for the identification of these SREs [9]. Some splicing motifs have been reported to be correlated with regulation of AS. For example, Holste et al. had provided a computational framework to identify splicing motifs and to predict AS events [10]. Enhancer had also been reported to correlate to AS changes [11–13].

Enhancer is a *cis*-regulatory element known as its characteristics: high abundance in genome, regulating genes in highly variable location, and lack of discriminative DNA sequence [14]. Enhancers have been demonstrated to physically interact with promoter and polymerase during transcription elongation [15, 16]. This physical interaction shortens the distance between enhancer and gene body, and further grants enhancers an opportunity to influence AS. Previous studies had demonstrated that enhancer can affect alternative splicing. For example, the insertion of the SV40 transcriptional enhancer is capable of inhibition of inclusive form of fibronectin extra domain I [11]. Another one example is that the downstream enhancer of protocadherin alpha can loop back to bind with promoter by coupling of CTCF and further affect AS [17]. These studies had shown that enhancer is capable of affecting AS events.

**Fig. 1:**
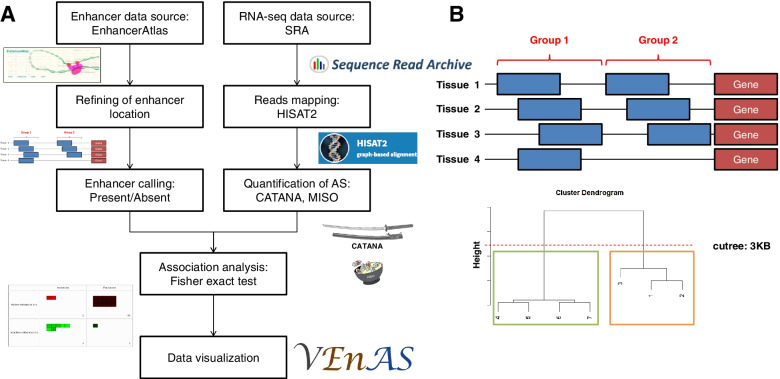
(**A**)Workflow or the analysis pipeline for identification of enhancer-AS associations. In the top left part of the analysis pipeline, we focused on enhancer datasets polish, including position refining and presence/absence calling. In the top right part, we focused on the processes for quantification of AS changes. We then conducted association analysis to identify enhancer-AS associations, and finally constructed a website called VEnAS for data visualization. (**B**) An example of refining enhancers between different tissues and cell lines.The blue boxes are representing to enhancers in different tissues or cell lines. The middle positions of enhancers are used for hierarchical clustering with centroid method. The cuttree threshold is set as 3 kilo bases. The green and orange boxes are representing to the two refined groups under the threshold

A previous study suggested that most of enhancers are inactive (poised) until the proper factor binds on it [18]. Thus, it is challenging for biologists to design a high-throughput experiment to identify the enhancer-AS associations. Because there is no genome-wide study to identify the associations, in this context, we developed a bioinformatics pipeline (Fig. 1A) to find out the significant enhancer-AS associations on a genome-wide scale by analyzing large amount of human and mouse transcriptomes. We further constructed a platform entitled VEnAS (Visualization of Enhancer-associated Alternative Splicing) to present the enhancer-AS associations.

## Methods

### Data selection and preparation

We downloaded enhancer datasets which include 28 human and 24 mouse tissues and cell lines from enhancerAtlas [19]. These tissues and cell lines were chosen because they have at least three paired RNA-seq datasets for quantification of AS. To prevent the data imbalance, we down-sampled the number of RNA-seq datasets to three. We then downloaded the chosen 84 human (28*3) and 72 mouse (24*3) RNA-seq fastq files from Sequence Read Archive (SRA) [20]. These fastq files were mapped onto the latest genome (GRCh38 for human and GRCm38 for mouse) by HISAT2 [21] with default parameters.

### Enhancer calling

The boundaries of enhancer could be incongruent due to tissue characteristics, enhancer calling methodologies, or batch effects from input data sets. Thus, refining the location of enhancers between different tissue types is required to eradicate the incongruence. To refine the enhancers between different tissues and cell lines, we took advantage of agglomerative hierarchical clustering with centroid method (Fig. 1B). We used the central position of each enhancer as input for hierarchical clustering. Previous studies had reported that the length of enhancer is ranged between 2–4 kilo bases [18, 22–24]. Thus, we set 3 kilo bases as a threshold to limit the growth of the clusters. After refining the location of enhancer, we were able to call the present or absent of enhancer between different tissues based on whether there is any enhancer located in the refined range.

### Quantification and categorization of AS

The v94 human and mouse genome annotations were downloaded from Ensembl. CATANA [25] was used upon the human and mouse genome annotation to obtain the latest version of AS annotation. The latest AS annotation and the mapped bam files (from data preparation) were used for MISO [26] to compute percent splice in (PSI), which is an inclusion index based on the number of junction reads [27]. The equation of PSI is defined as

$${\text{PSI}} \, \text{=} \, \frac{\text{Junction reads supporting to inclusive form}}{\left(\text{Junction reads supporting to inclusive form}\text{ + Junction reads supporting to exclusive form}\right)}$$

To guarantee the AS changes of a given AS event from human 84 or mouse 72 samples are large enough, we removed the AS events with the PSI range across all samples less than 0.1. After that, we conducted Z-transformation upon all PSI values across tissues to capture the changes of a given AS between tissues. To categorize whether a tissue does have an AS change, the tissue having Z-transformed PSI value (Z-PSI) larger than 1 is defined as “inclusive shift”, while the tissue having Z-PSI smaller than -1 is defined as “exclusive shift”.

### Association analysis

With the labels present/absent of enhancers and inclusive/exclusive shift of AS changes, for each enhancer-AS pair we can generate a two-by-two contingency table containing the number of samples in the four cells. We removed enhancer-AS pairs having low strength of association in the contingency table to improve the precision of the association analysis and reduce the false results. Thus, we only included the enhancer-AS pairs for analysis in which the odds ratio must be larger than 2 or less than 0.5 accompanied by the effective size constrain (the number difference between concordant and discordant cells must be larger than 10). Then the Fisher exact test was conducted exhaustively throughout all the enhancer-AS pairs to calculate the *p*-value. All the *p*-values were then adjusted by Benjamini–Hochberg procedure false discovery rate (FDR) to obtain *q*-values. An enhancer-AS association was considered significant if the *q*-value is smaller than 0.05.

### Implementation of VEnAS

The VEnAS database was written by a combination of Perl, Python, and R for data processing and statistical analysis. The web server of VEnAS was implemented with a combination of PHP, Google Polymer framework, and MySQL on Ubuntu server. For efficiently storing and querying, the analysis result and other integrated data were subjected to database normalization. The schema of the normalized MySQL database table is shown in the Figure S1. The tables holding PSI and genomic location of enhancer were separated for parallel querying by MySQL. In addition, the table holding index for autocompletion during user query is shown on the top-left side of Figure S1. The keywords used for constructing index include Ensembl gene accession, gene symbol, and gene description.

## Results

To identify the enhancer-AS associations on a genome-wide scale, we developed an analysis pipeline (Fig. 1A, detailed in Methods). We first curated the enhancer profiles and RNA-seq datasets of 28 human and 24 mouse tissues and cell lines for analysis. Since the profile of active enhancer is naturally varied between different tissues and cell lines [28], we refined the boundaries of enhancers and generated enhancer calling using the hierarchical clustering method. We then used CATANA and MISO to quantify and categorize AS events from RNA-seq datasets. To further check the similarity or overlapping event between different samples, we computed the Jaccard coefficient index (Figure S2). The result shows that the enhancer-AS events are quite similar within the triplicated samples under the same tissue type but different between tissues. The Fisher exact test was performed to identify the significant enhancer-AS associations with present/absent of enhancer and inclusive/exclusive shift of AS types.

### Enhancer-AS associations in human and mouse

**Fig. 2:**
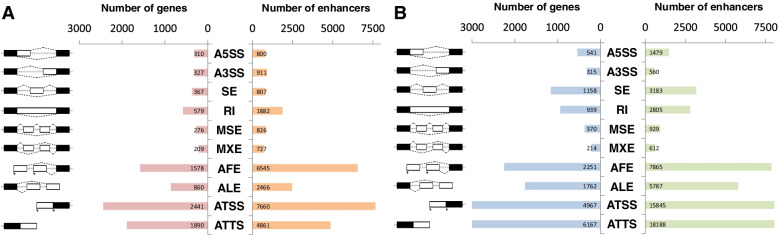
The counting number of genes and enhancer associated to AS changes in (**A**) human and (**B**) mouse. The number of genes having significant (FDR *q*-value < 0.05) enhancer-associated AS and the number of enhancers significantly (FDR *q*-value < 0.05) associated to AS changes are shown in x-axis. All ten types of AS are counted independently. Every gene having multiple significant events is counted once. The ten types of AS are including alternative 5’/3’ splice site (A5SS, A3SS), skipped exon (SE), retained intron (RI), multiple skipped exons (MSE), mutually exclusive exons (MXE), alternative first/last exons (AFE, ALE), and alternative transcription start/termination sites (ATSS, ATTS)

By conducting association analysis with absent/present of enhancer and inclusive/exclusive shift of AS event, we found that 3,242 human genes and 7,716 mouse genes have at least one significant enhancer-AS association, and 11,262 human enhancers and 26,083 mouse enhancers are participating in AS changes (Table 1 and Table 2). Previous study had mentioned that transcripts having alternative start and termination sites shape the major transcriptome diversity across human tissues [13]. As expected, in our results, the numbers of genes having associations between enhancers and the AS types regarding alternative transcription initiation and termination sites (AFE, ALE, ATSS and ATTS) are notably higher than the six canonical AS types (A5SS, A3SS, SE, RI, MSE, MXE) in human and mouse (Fig. 2).

**Table 1:** The counting table of human genes and enhancer having enhancers having enhancer-AS association for different AS types. The row “All” represents the number of genes or enhancers having associations in any types of AS. The column “input” means the number of genes or enhancers which are qualified for the analysis. The column “significant” represents the number of genes or enhancers pass the *q*-value smaller than 0.05

AS type		Counting by genes				Counting by enhancers		
		Significant	Input	Percentage		Significant	Input	Percentage
A5SS		310	732	42.35%		800	4542	17.61%
A3SS		327	724	45.17%		911	4727	19.27%
SE		367	1406	26.10%		807	8610	9.37%
RI		579	977	59.26%		1882	6896	27.29%
MSE		276	629	43.88%		826	4267	19.36%
MXE		209	309	67.64%		727	1757	41.38%
AFE		1578	2121	74.40%		6545	15,695	41.70%
ALE		860	1858	46.29%		2466	13,137	18.77%
ATSS		2441	3548	68.80%		7660	25,654	29.86%
ATTS		1890	4011	47.12%		4861	29,766	16.33%
All		3242	4658	69.60%		11,262	35,158	32.03%

**Table 2:** The counting table of mouse genes and enhancer having enhancer-AS association for different AS types. The row “All” represents the number of genes or enhancers having associations in any types of AS. The column “input” means the number of genes or enhancers which are qualified for the analysis. The column “significant” represents the number of genes or enhancers pass the *q*-value smaller than 0.05

AS type		Counting by genes				Counting by enhancers		
		Significant	Input	Percentage		Significant	Input	Percentage
A5SS		541	689	78.52%		1479	2984	49.56%
A3SS		315	726	43.39%		560	2969	18.86%
SE		1158	1530	75.69%		3183	6319	50.37%
RI		939	1210	77.60%		2805	5942	47.21%
MSE		370	484	76.45%		929	1838	50.54%
MXE		214	247	86.64%		612	986	62.07%
AFE		2251	2643	85.17%		7865	14,387	54.67%
ALE		1762	2170	81.20%		5787	11,067	52.29%
ATSS		4967	5593	88.81%		15,845	27,158	58.34%
ATTS		6167	7158	86.16%		18,188	36,008	50.51%
All		7716	8429	91.54%		26,083	45,810	56.94%

Gene and enhancer are many-to-many relationship [29]. One given gene could be associated to multiple enhancers, and vice versa. Here we would like to know that under consideration of association with AS changes, how many enhancers are associated to one given gene and how many genes are associated to one given enhancer. We further interrogated the association relationship between enhancer and genes by examining the number of enhancers per gene (also the genes per enhancer). According to the annotation from enhancerAtlas, on average, each gene is paired with 60.32 enhancers in human and 68.47 enhancers in mouse. Our association analysis suggests that given one gene, on average, 3.88 of the 60.32 enhancers (6.43%) in human and 3.54 of the 68.47 enhancers (5.17%) in mouse are associated to AS change (Figure S3A and S3B). For enhancers, on average each one enhancer is paired to 7.66 genes in human and 9.29 genes in mouse according to enhancerAtlas, but in our result one enhancer is significantly associated to AS change with only 1.28 genes human and 1.22 genes in mouse (Figure S3C and S3D).

### Investigations of the genetic properties of identified enhancer-AS associations

To further understand the genetic properties of identified enhancer-AS associations, we observed the proportion of enhancer-AS associations which have both homologous genes and homologous enhancers between human and mouse. For each gene in human, we defined its homolog in mouse according to the homologs list provided in Mouse Genome Informatics (MGI) [30]. For each enhancer in human, we obtained its homologous enhancers in mouse by conducting the CrossMap [31] with the human and mouse chain file and, which is the pairwise alignment between two reference assemblies from Ensembl [32]. We found that about 52% of the significant and 35% of the insignificant enhancer-AS associations in human have homologous genes accompanied with homologous enhancers in mouse (Table 3). The Welch two sample t-test shows significant difference (*p*-value = 5.56 × 10^–11^) upon percentages of significant enhancer-AS pairs with homologous genes and enhancers in all ten types of AS against insignificant groups. This suggests that the significant enhancer-AS pairs are more likely to be the co-existence of homologous genes and homologous enhancers than insignificant enhancer-AS pairs. Similar trends with lower percentages were found when we check the significant enhancer-AS pairs (Welch two sample t-test *p*-value = 1.906 × 10^–13^) in mouse (Table 4). These results show that the significant enhancer-AS associations we identified are more likely to be the co-existence of homologous genes accompanied with homologous enhancers in both human and mouse rather than conservation of enhancer sequence only.

**Table 3:** The counting table of human significant and insignificant enhancer-AS pairs accompanied with both homologous enhancers. The numbers of total significant enhancer-AS pairs, significant enhancer-AS pairs with homologous genes and enhancers, total insignificant enhancer-AS pairs, insignificant enhancer-AS pairs with homologous genes or enhancers in all ten types of AS are provided. The percentages of enhancer-AS pairs with homologous genes and enhancers in all ten types of AS are calculated

AS type		Significant pairs	with homologous genes and enhancers	Percentage		Insignificant pairs	with homologous genes and enhancers	Percentage
A5SS		1046	553	52.87%		266,860	97,639	36.59%
A3SS		2129	1122	52.70%		436,986	160,042	36.62%
SE		3070	1631	53.13%		731,628	256,762	35.09%
RI		5792	3115	53.78%		894,218	319,572	35.74%
MSE		6980	3681	52.74%		930,339	329,901	35.46%
MXE		7950	4124	51.87%		944,441	332,218	35.18%
AFE		38,481	18,452	47.95%		3,672,503	1,235,971	33.65%
ALE		45,132	21,118	46.79%		4,134,137	1,377,293	33.32%
ATSS		92,545	47,260	51.07%		11,645,531	4,181,752	35.91%
ATTS		113,447	59,169	52.16%		12,675,728	4,528,278	35.72%

**Table 4:** The counting table of mouse significant and insignificant enhancer-AS pairs accompanied with both homologous genes and homologous enhancers

AS type		Significant pairs	with homologous genes and enhancers	Percentage		Insignificant pairs	with homologous genes and enhancers	Percentage
A5SS		1648	558	33.86%		387,866	106,854	27.55%
A3SS		2232	787	35.26%		696,666	196,317	28.18%
SE		5963	1975	33.12%		1,138,626	308,850	27.12%
RI		9662	3363	34.81%		1,518,594	421,336	27.75%
MSE		10,670	3674	34.43%		1,552,868	429,533	27.66%
MXE		11,276	3883	34.44%		1,556,723	430,275	27.64%
AFE		32,678	10,732	32.84%		3,796,988	985,494	25.95%
ALE		44,033	14,500	32.93%		4,133,025	1,067,346	25.82%
ATSS		100,364	34,303	34.18%		12,505,889	3,380,938	27.03%
ATTS		164,111	56,071	34.17%		13,450,641	3,630,266	26.99%

### Visualization of enhancer-AS associations

**Fig. 3:**
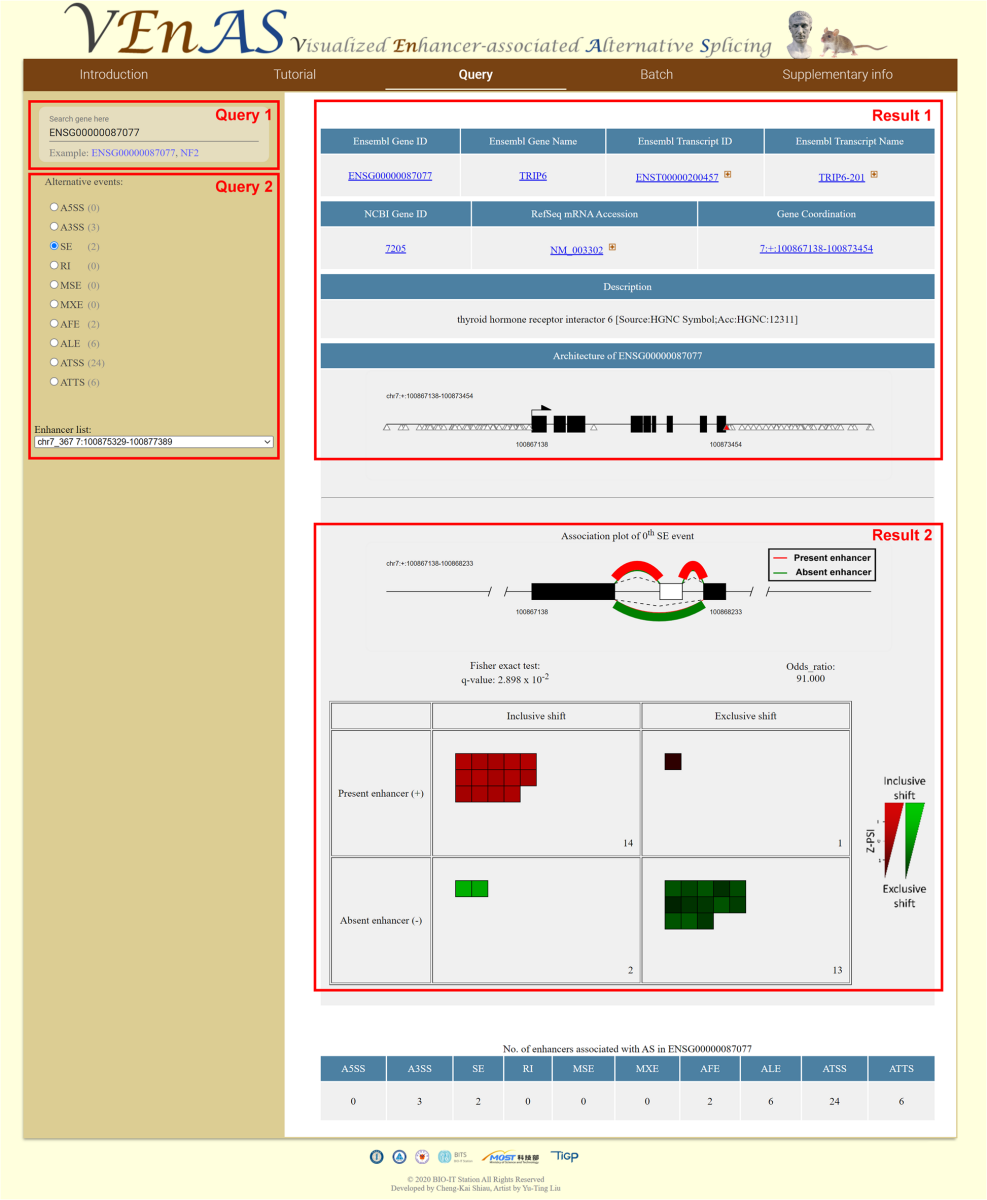
The webpage of VEnAS and query steps. Following by the queries (e.g. Query 1 and 2), users can obtain gene architecture, association plot, and detailed statistical information (e.g. Result 1 and 2) of VEnAS database conveniently

To visualize the enhancer-AS associations, we constructed a platform named VEnAS. VEnAS provides intuitive genomic architecture, association plot, and contingency table of all the significant enhancer-AS associations (Fig. 3). To query VEnAS, users can input Ensembl gene ID or gene symbol (Query 1 in Fig. 3). The auto-completion function would help users find out the gene of interests. The web server provides portable gene information for convenient linking to Ensembl, NCBI, and RefSeq (Result 1 in Fig. 3). After users select an AS type and a corresponding enhancer (Query 2 in Fig. 3), VEnAS shows the architecture of the gene with enhancer, association plot, and a two-by-two contingency table (Query 2 in Fig. 3). For splicing display, the bending curve drawn above exons represents the inclusive form of AS products, while the curve drawn below exons represents the exclusive form. The width of curves represents the number of biological replicates which support the association events. Moreover, the colors denote whether enhancer is active or inactive. In the top of a two-by-two contingency table, the FDR adjusted *q*-value of the Fisher exact test and the odds ratio are also provided. Inside the table, the color boxes are representing biological replicates having AS shifted to inclusion or exclusion. The color intensity of the boxes is proportional to the Z-PSI. The tissue name and Z-PSI would be displayed when the mouse cursor is hovering atop the box. Additionally, VEnAS provides batch retrieval function. The user could send a list of Ensembl gene ID(s) obtained from any other analysis tool or software in the batch retrieval web page through pasting in dialog box or uploading file. VEnAS can convert the visualized results into PDF file format for users for further analyses.

## Case study

**Fig. 4:**
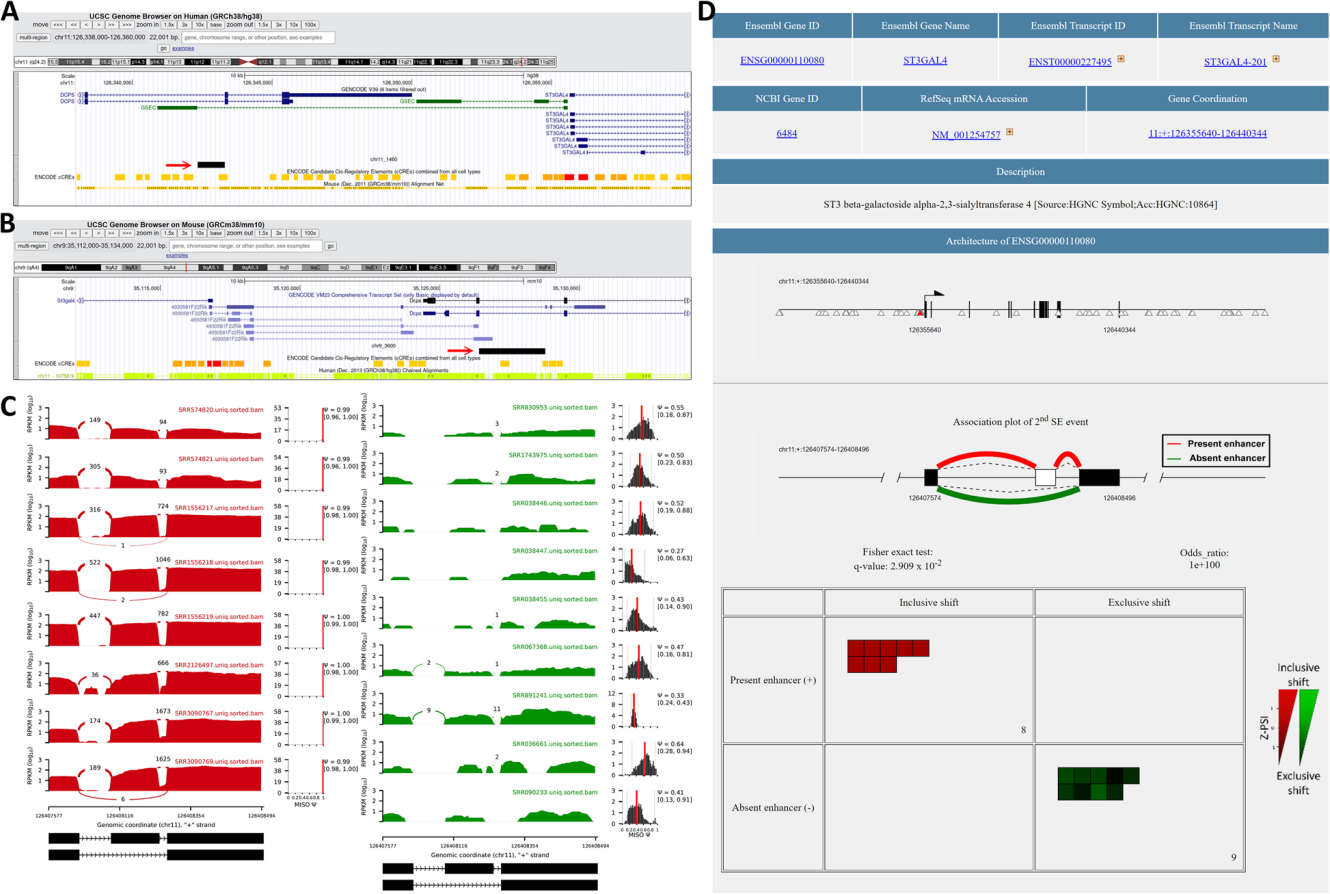
A real case for the enhancer-AS association. The associated enhancers located in upstream of human gene ST3GAL4 (**A**) and mouse gene St3gal4 (**B**) marked by the red arrows. The tracks showing ENCODE *cis*-regulatory elements and genomic synteny between human and mouse are provided in the below. **C** The sashimi plots and PSI histograms of human ST3GAL4 2^nd^ skipped exon (SE) event in 17 samples. The plots in red indicate that the samples having present associated enhancer chr11_1460, while the plots in green indicating the absence of chr11_1460. **D** The VEnAS result of 2^nd^ SE event in ENSG00000110080 and associated enhancer chr11_1460. The location of enhancer indicated by red triangle in genomic architecture, association plot, and two-by-two contingency table are provided

We have identified lots of enhancer-AS associations in this study. However, it is difficult to find out large scale biological evaluation or literature evidence. Hence, we performed comparative genomics analysis between human and mouse as well as observed the splicing events to further evaluate the identified associations. Below is a case demonstrating the robustness of our finding. In Manduchi et al*.*’s study [33], they identified 35 significant SNP marks and enhancers which are associated to Type 2 diabetes with combination of epigenomic markers and genome wide association studies (GWAS). In their result, gene ST3GAL4 is associated to two SNP markers located within an enhancer which is named chr11_1460 in our system. As shown in Fig. 4A, the enhancer is marked by ENCODE as a *cis*-regulatory element in human. As shown in comparative genomics data track, the genomic region of this enhancer is located within synteny between human and mouse. In mouse, the associated enhancer is named chr9_3600, which is also marked as a *cis*-regulatory element by ENCODE and within the syntenic region shared with human enhancer chr11_1460 (Fig. 4B). Furthermore, we utilized MISO to draw sashimi plots and PSI histograms [26] to illustrate that the presence/absence of the associated enhancer is associated to skipped exon event (SE) of ST3GAL4 in human (Fig. 4C). The PSI histograms show that all the PSI values are closed to “1”, *i.e.* the inclusive form dominated, in the samples where the enhancer is present. On the contrary, the PSI values are decreased to about 0.5 in the samples when the enhancer is absent. The strength of association between enhancer and SE is significant ($q-\mathrm{value}=2.909\mathrm{ x }{10}^{-2}$, as shown in Fig. 4D). Taking together the literature evidence, comparative genomics data, and PSI distribution; we did successfully demonstrate the existence of the enhancer-AS association.

## Discussions

Previous studies showed that some enhancers are conserved between human and mouse [34] while some enhancers might be reprogrammed after human-mouse speciation [35]. To investigate whether the enhancers associated to AS existed in human and mouse are conserved or not, we further examined the conservation score difference between significant and insignificant enhancers. The conservation score of enhancer sequence between human and mouse were downloaded from Ensembl v94 compara 32 amniotes datasets [36]. After comparison, we didn’t find any difference of conservation score of enhancer sequence between significant and insignificant enhancer-AS associations (data not shown).

As we already know that enhancers could also serve as a hub for binding of transcription factors [18], we tried to annotate known motifs on enhancer regions by DREME [37] and TomTom [38] with position frequency matrix from JASPAR [39]. However, we didn’t find any differentially enriched known motifs shared between human and mouse. Though we didn’t find any advanced evidence, more data sets are required to conclude that the enhancers associated to AS are newly emerged or reprogrammed after human-mouse speciation.

In 2013, a new concept of super-enhancer had been proposed [40, 41]. Super-enhancers are considered to be a cluster of several different enhancers with exceptional higher binding of transcriptional coactivators [40, 41]. Super-enhancers are usually longer than typical enhancers, with a median length of 8.7 kb [42]. Recently, more and more super-enhancer databases about super-enhancer characteristics and associated genes are available, such as dbSUPER [43], SEdb [44], and SEA [45]. It has been reported that super-enhancer is capable of regulating alternative splicing in smooth muscle [46]. However, our current statistical analysis method is designed for one enhancer on one AS event rather than multiple/combinatorial enhancers on one AS event. To pin-point the correlation between the combination of transcription factors and AS events requires a more sophisticated method. In the future, we will pursue a genome-wide method to reveal the correlation between super-enhancer and alternative splicing event.

## Conclusion

In this study, an analysis pipeline to identify enhancer-AS associations was proposed. We included 84 RNA-seq data sets across 28 tissues and cell lines in human and 72 RNA-seq data sets across 24 tissues and cell lines in mouse for analysis. In total, 3,242 human genes and 7,716 mouse genes having at least one significant enhancer-AS were identified. On average, about 5–6% of the enhancers of one given gene are associated to AS change, and one given enhancer is associated to 1.28 human or 1.22 mouse genes. The significant enhancer-AS associations are more likely to be the co-existence of homologous genes and homologous enhancers in both human and mouse. Finally, we constructed VEnAS to provide comprehensive enhancer-associated AS results for scientists, including genomic architecture, intuitive association plot, and contingency table. We believe that our study is helpful in further understanding the roles of enhancers on regulating alternative splicing.

## Supplementary Information

**Additional file 1: Figure S1. **Thedetailed schema of data warehousing in MySQL. All the data tables includingcolumn names are illustrated. The primary keys for linking tables are depictedwith black lines. **Figure S2. **Thecomparison of sample similarity in skipped exon (SE).The Jaccardcoefficient index is pair-wisely computed to present the enhancer-AS similarityor overlapping between different samples. The number of enhancer-AS eventswhich have identical enhancer present/absent calling and the sameinclusive/exclusive AS shift are calculated, and then divided by the totalenhancer-AS events to compute the Jaccard coefficient index. The result showsthat the enhancer-AS events are different between tissues but quite similarwithin the triplicated samples under the same tissue type, except fetal stomachSRR980482 which has the lowest Jaccard index score comparing to the other twofetal stomach samples.

## Abbreviations

A3SS: Alternative 3’ splice site
A5SS: Alternative 5’ splice site
AFE: Alternative first exon
ALE: Alternative last exon
AS: Alternative splicing
ATSS: Alternative transcription start site
ATTS: Alternative transcription termination site
enhancer-AS: Association between enhancer and alternative splicing
FDR: Benjamini–Hochberg false discovery rate
GWAS: Genome wide association study
MSE: Multiple skipped exon
MXE: Mutually exclusive exon
PSI: Percent splice in
RI: Retained intron
SE: Skipped exon
SNP: Single nucleotide polymorphism
SRE: Splicing regulatory elements
Z-PSI: Z-transformed PSI

## Declarations

### Availability of Data and Materials

All the raw datasets are downloaded from enhancerAtlas (http://www.enhanceratlas.org) and SRA (https://www.ncbi.nlm.nih.gov/sra/). The tissues and cell lines datasets used for the analysis are listed on the VEnAS web site (http://venas.iis.sinica.edu.tw/). The analysis result spreadsheets are available on VEnAS.

All the analysis pipeline and data source for analysis are available on GitHub: https://github.com/shiauck/VEnAS.

## References

[1] Pan Q, Shai O, Lee LJ, Frey BJ, Blencowe BJ (2008). Deep surveying of alternative splicing complexity in the human transcriptome by high-throughput sequencing. Nat Genet.

[2] Kriventseva EV, Koch I, Apweiler R, Vingron M, Bork P, Gelfand MS, Sunyaev S (2003). Increase of functional diversity by alternative splicing. Trends Genet.

[3] Weyn-Vanhentenryck SM, Feng H, Ustianenko D, Duffié R, Yan Q, Jacko M (2018). Precise temporal regulation of alternative splicing during neural development. Nat Commun.

[4] Yeo G, Holste D, Kreiman G, Burge CB (2004). Variation in alternative splicing across human tissues. Genome Biol.

[5] Noh SJ, Lee K, Paik H, Hur CG (2006). TISA: Tissue-specific Alternative Splicing in Human and Mouse Genes. DNA Res.

[6] Planells B, Gómez-Redondo I, Pericuesta E, Lonergan P, Gutiérrez-Adán A (2019). Differential isoform expression and alternative splicing in sex determination in mice. BMC Genomics.

[7] Gibilisco L, Zhou Q, Mahajan S, Bachtrog D (2016). Alternative Splicing within and between *Drosophila* Species, Sexes, Tissues, and Developmental Stages. PLoS Genet..

[8] Foret S, Kucharski R, Pellegrini M, Feng S, Jacobsen SE, Robinson GE (2012). DNA methylation dynamics, metabolic fluxes, gene splicing, and alternative phenotypes in honey bees. Proc Natl Acad Sci U S A.

[9] Wang Y, Wang Z (2014). Systematical identification of splicing regulatory *cis*-elements and cognate *trans*-factors. Methods.

[10] Holste D, Ohler U (2008). Strategies for Identifying RNA Splicing Regulatory Motifs and Predicting Alternative Splicing Events. PLoS Comput Biol..

[11] Kadener S, Fededa JP, Rosbash M, Kornblihtt AR (2002). Regulation of alternative splicing by a transcriptional enhancer through RNA pol II elongation. Proc Natl Acad Sci U S A.

[12] Esumi S, Kakazu N, Taguchi Y, Hirayama T, Sasaki A, Hirabayashi T (2005). Monoallelic yet combinatorial expression of variable exons of the protocadherin-alpha gene cluster in single neurons. Nat Genet.

[13] Reyes A, Huber W (2018). Alternative start and termination sites of transcription drive most transcript isoform differences across human tissues. Nucleic Acids Res.

[14] Pennacchio LA, Bickmore W, Dean A, Nobrega MA, Bejerano G (2013). Enhancers: five essential questions. Nat Rev Genet.

[15] Lee K, Hsiung CCS, Huang P, Raj A, Blobel GA (2015). Dynamic enhancer–gene body contacts during transcription elongation. Genes Dev.

[16] Schoenfelder S, Fraser P (2019). Long-range enhancer-promoter contacts in gene expression control. Nat Rev Genet.

[17] Ong CT, Corces VG (2014). CTCF: An Architectural Protein Bridging Genome Topology and Function. Nat Rev Genet.

[18] Buecker C, Wysocka J (2012). Enhancers as information integration hubs in development: lessons from genomics. Trends Genet.

[19] Gao T, He B, Liu S, Zhu H, Tan K, Qian J (2016). EnhancerAtlas: a resource for enhancer annotation and analysis in 105 human cell/tissue types. Bioinformatics.

[20] Leinonen R, Sugawara H (2011). The Sequence Read Archive. Nucleic Acids Res.

[21] Kim D, Paggi JM, Park C, Bennett C, Salzberg SL (2019). Graph-based genome alignment and genotyping with HISAT2 and HISAT-genotype. Nat Biotechnol.

[22] Kim TK, Hemberg M, Gray JM, Costa AM, Bear DM, Wu J (2010). Widespread transcription at neuronal activity-regulated enhancers. Nature.

[23] Podsiadło A, Wrzesień M, Paja W, Rudnicki W, Wilczyński B (2013). Active enhancer positions can be accurately predicted from chromatin marks and collective sequence motif data. BMC Syst Biol.

[24] Whalen S, Truty RM, Pollard KS (2016). Enhancer-promoter interactions are encoded by complex genomic signatures on looping chromatin. Nat Genet.

[25] Shiau CK, Huang JH, Tsai HK (2019). CATANA: a tool for generating comprehensive annotations of alternative transcript events. Bioinformatics.

[26] Katz Y, Wang ET, Airoldi EM, Burge CB (2010). Analysis and design of RNA sequencing experiments for identifying isoform regulation. Nat Methods.

[27] Venables JP, Klinck R, Bramard A, Inkel L, Dufresne-Martin G, Koh C (2008). Identification of alternative splicing markers for breast cancer. Cancer Res.

[28] Shen Y, Yue F, McCleary DF, Ye Z, Edsall L, Kuan S (2012). A map of the *cis*-regulatory sequences in the mouse genome. Nature.

[29] Fishilevich S, Nudel R, Rappaport N, Hadar R, Plaschkes I, Stein TI, *et al*. GeneHancer: genome-wide integration of enhancers and target genes in GeneCards. Database. 2017;2017:bax028. 10.1093/database/bax028 PMC5467550 28605766

[30] Bult CJ, Blake JA, Smith CL, Kadin JA, Richardson JE (2019). the Mouse Genome Database Group. Mouse Genome Database (MGD) 2019. 2019. Nucleic Acids Res.

[31] Zhao H, Sun Z, Wang J, Huang H, Kocher JP, Wang L (2014). CrossMap: a versatile tool for coordinate conversion between genome assemblies. Bioinformatics.

[32] UCSC chain file from hg19 (GRCh37) to mm9 (GRCm37). http://hgdownload.soe.ucsc.edu/goldenPath/hg19/liftOver/hg19ToMm9.over.chain.gz. Accessed 21 Sep 2020.

[33] Manduchi E, Williams SM, Chesi A, Johnson ME, Wells AD, Grant SFA (2018). Leveraging epigenomics and contactomics data to investigate SNP pairs in GWAS. Hum Genet.

[34] Vilar D, Berthelot C, Aldridge S, Rayner TF, Lukk M, Pignatelli M (2015). Enhancer evolution across 20 mammalian species. Cell.

[35] Flores MA, Ovcharenko I (2018). Enhancer reprogramming in mammalian genomes. BMC Bioinformatics.

[36] Cooper GM, Stone EA, Asimenos G, Batzoglou S, NISC Comparative Sequencing Program, Green ED (2005). Distribution and intensity of constraint in mammalian genomic sequence. Genome Res.

[37] Bailey TL (2011). DREME: motif discovery in transcription factor ChIP-seq data. Bioinformatics.

[38] Gupta S, Stamatoyannopoulos JA, Bailey TL, Noble WS (2007). Quantifying similarity between motifs. Genome Biol.

[39] Fornes O, Castro-Mondragon JA, Khan A, Lee RVD, Zhang X, Richmond PA (2020). JASPAR 2020: update of the open-access database of transcription factor binding profiles. Nucleic Acids Res.

[40] Whyte WA, Orlando DA, Hnisz D, Abraham BJ, Lin CY, Kagey MH (2013). Master Transcription Factors and Mediator Establish Super-Enhancers at Key Cell Identity Genes. Cell.

[41] Hnisz D, Abraham BJ, Lee TI, Lau A, Saint-Andre V, Sigova AA, et al. Transcriptional super-enhancers connected to cell identity and disease. Cell. 2013;155(4): 10.1016/j.cell.2013.09.053. 10.1016/j.cell.2013.09.053 PMC3841062 24119843

[42] Moorthy SD, Davidson S, Shchuka VM, Singh G, Malek-Gilani N, Langroudi L (2017). Enhancers and super-enhancers have an equivalent regulatory role in embryonic stem cells through regulation of single or multiple genes. Genome Res.

[43] Khan A, Zhang X (2016). dbSUPER: a database of super-enhancers in mouse and human genome. Nucleic Acids Res.

[44] Jiang Y, Qian F, Bai X, Liu Y, Wang Q, Ai B (2019). SEdb: a comprehensive human super-enhancer database. Nucleic Acids Res.

[45] Chen C, Zhou D, Gu Y, Wang C, Zhang M, Lin X (2020). SEA version 3.0: a comprehensive extension and update of the Super-Enhancer archive. Nucleic Acids Res.

[46] Nakagaki-Silva EE, Gooding C, Llorian M, Jacob AG, Richards F, Buckroyd A (2019). Identification of RBPMS as a mammalian smooth muscle master splicing regulator via proximity of its gene with super-enhancers. eLife.

