# Supplementary material for: Genome-wide identification of associations between enhancer and alternative splicing in human and mouse
Source: BMC Genomics. 2022 May 9;22(Suppl 5):919. doi: 10.1186/s12864-022-08537-1 (PMC9082955; doi:10.1186/s12864-022-08537-1)
Supplement: Supplementary file 1 — Additional file 1: Figure S1. Thedetailed schema of data warehousing in MySQL. All the data tables includingcolumn names are illustrated. The primary keys for linking tables are depictedwith black lines. Figure S2. Thecomparison of sample similarity in skipped exon (SE).The Jaccardcoefficient index is pair-wisely computed to present the enhancer-AS similarityor overlapping between different samples. The number of enhancer-AS eventswhich have identical enhancer present/absent calling and the sameinclusive/exclusive AS shift are calculated, and then divided by the totalenhancer-AS events to compute the Jaccard coefficient index. The result showsthat the enhancer-AS events are different between tissues but quite similarwithin the triplicated samples under the same tissue type, except fetal stomachSRR980482 which has the lowest Jaccard index score comparing to the other twofetal stomach samples. [file 12864_2022_8537_MOESM1_ESM.docx]

**
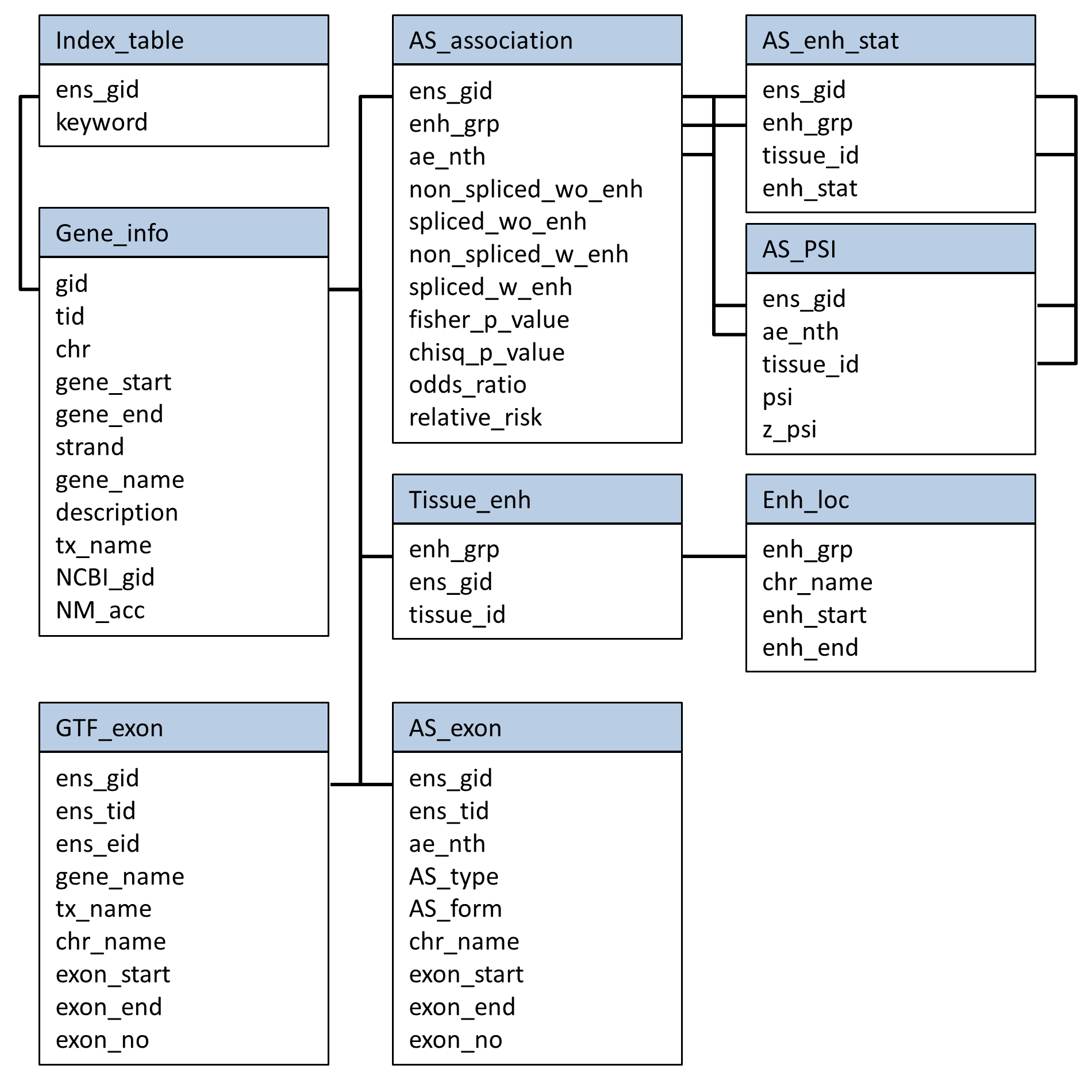
Figure S1. The detailed schema of data warehousing in MySQL.** All the data tables including column names are illustrated. The primary keys for linking tables are depicted with black lines.


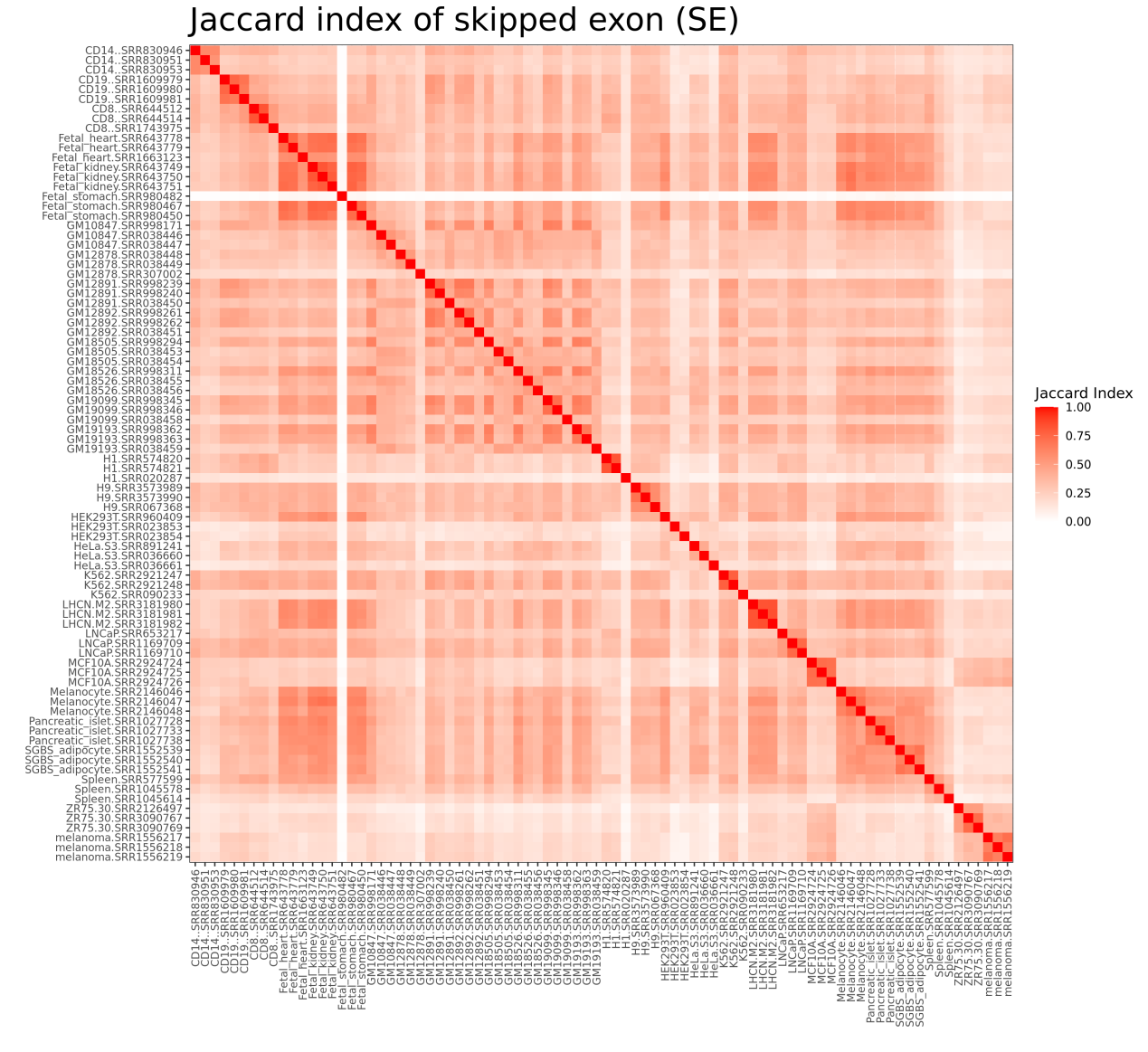


**Figure S2. The comparison of sample similarity in skipped exon (SE).** The Jaccard coefficient index is pair-wisely computed to present the enhancer-AS similarity or overlapping between different samples. The number of enhancer-AS events which have identical enhancer present/absent calling and the same inclusive/exclusive AS shift are calculated, and then divided by the total enhancer-AS events to compute the Jaccard coefficient index. The result shows that the enhancer-AS events are different between tissues but quite similar within the triplicated samples under the same tissue type, except fetal stomach SRR980482 which has the lowest Jaccard index score comparing to the other two fetal stomach samples.

**
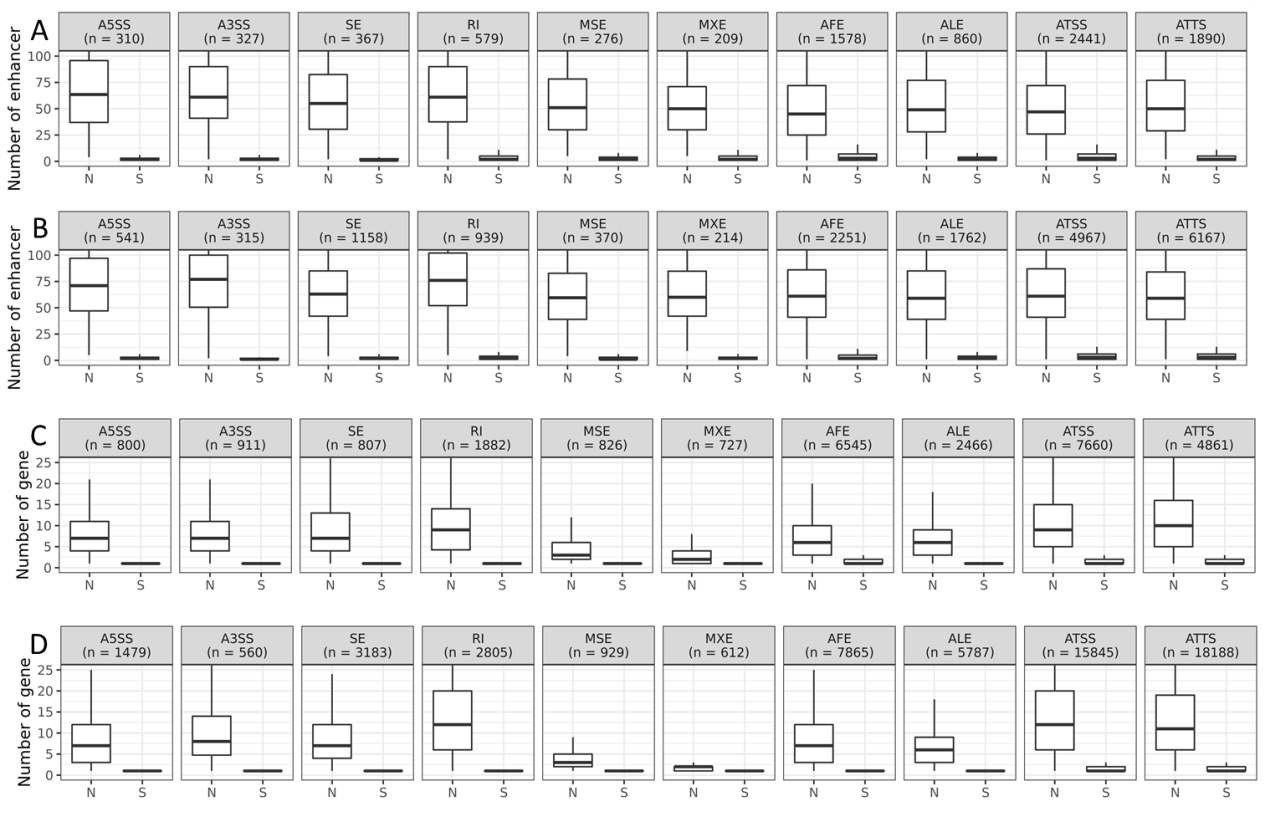
Figure S3. The association relationships in human and mouse.** The boxplot of enhancer per gene in (A) human and (B) mouse. In the x-axis, “N” denotes all the genes having at least one gene-enhancer pairs and passing our filtering criteria mentioned in methodology. “S” means the genes having significant enhancer-AS associations. The boxplot of genes per enhancer in (C) human and (D) mouse. “N” represents all the enhancers having at least one gene-enhancer pairs and passing our filtering criteria. “S” denotes the enhancers having significant enhancer-AS associations.
